# Low-Rank and Sparse Matrix Decomposition for Genetic Interaction Data

**DOI:** 10.1155/2015/573956

**Published:** 2015-07-26

**Authors:** Yishu Wang, Dejie Yang, Minghua Deng

**Affiliations:** ^1^Center for Quantitative Biology, Peking University, Beijing 100871, China; ^2^Institute of Computing Technology, Chinese Academy of Science, Beijing 100190, China; ^3^School of Mathematical Sciences, Peking University, Beijing 100871, China; ^4^Center for Statistical Sciences, Peking University, Beijing 100871, China

## Abstract

*Background*. Epistatic miniarray profile (EMAP) studies have enabled the mapping of large-scale genetic interaction networks and generated large amounts of data in model organisms. One approach to analyze EMAP data is to identify gene modules with densely interacting genes. In addition, genetic interaction score (*S* score) reflects the degree of synergizing or mitigating effect of two mutants, which is also informative. Statistical approaches that exploit both modularity and the pairwise interactions may provide more insight into the underlying biology. However, the high missing rate in EMAP data hinders the development of such approaches. To address the above problem, we adopted the matrix decomposition methodology “low-rank and sparse decomposition” (LRSDec) to decompose EMAP data matrix into low-rank part and sparse part. *Results*. LRSDec has been demonstrated as an effective technique for analyzing EMAP data. We applied a synthetic dataset and an EMAP dataset studying RNA-related processes in *Saccharomyces cerevisiae*. Global views of the genetic cross talk between different RNA-related protein complexes and processes have been structured, and novel functions of genes have been predicted.

## 1. Introduction

Genetic interactions, which represent the degree to which the presence of one mutation modulates the phenotype of a second mutation, could be measured systematically and quantitatively in recent years [1, 2]. Genetic interactions can reveal functional relationships between genes and pathways. Furthermore, genetic networks measured via high-throughput technologies could reveal the schematic wiring of biological processes and predict novel functions of genes [3]. Recently, several high-throughput technologies have been developed to identify genetic interactions at genome scale, including Synthetic Genetic Array (SGA) [4], Diploid-Based Synthetic Lethality Analysis on Microarrays (dSLAM) [5], and epistatic miniarray profile (EMAP) [6]. In particular, EMAP systematically construct double deletion strains by crossing query strains with a library of test strains and identify genetic interactions by measuring a growth phenotype. An *S* score was calculated based on statistical methods for each pair of genes, while negative *S* scores represent synthetic sick/lethal and positive *S* scores indicate alleviating interactions [6].

Consequently, for each pair of genes, there are two different measures of relationship in EMAP platform. First, the genetic interaction score (*S* score) represents the degree of synergizing or mitigating effects of the two mutations in combination. Second, the similarity (typically measured as a correlation) of their genetic interaction profiles represents the congruency of the phenotypes of the two mutations across a wide variety of genetic backgrounds. So there are two important aspects in exploiting EMAP data. On the one hand, cellular functions and processes are carried out in series of interacting events, so genes participating in the same biological process tend to interact with each other. Therefore, algorithms that detect gene modules composed of densely interacting genes are of great interest. Within these modules, genes tend to have similar genetic interaction profiles; thus the submatrix for these genes tends to have a low-rank structure. On the other hand, the cross talks between modules are usually indicated by gene pairs with high *S* scores (so that the genetic interaction is significant). Removing them results in better low-rank structure. Evocatively, these gene pairs are likely shadows over the low-rank matrix and connect different rank areas. These cross talks reveal the relationships of different biological process or protein complexes. Meanwhile, gene pairs exhibiting high absolute value of *S* scores may encode proteins that are physically associated or be enriched in protein-protein interactions [7–9]. So the investigation of *S* score is equally important. However, the current methodologies in genetic interaction networks analysis did not efficiently address these two important issues simultaneously.

In order to identify modules and between-module cross talks in genetic interaction networks, we employ the “low-rank and sparse decomposition” (LRSDec) to decompose EMAP data matrix into a low-rank part and a sparse part. We propose that the low-rank structure accounts for gene modules, in which genes have high correlations, and the sparsity matrix captures the significant *S* scores. In particular, entries in sparse matrix found by LRSDec correspond to two sources of biologically meaningful interactions, within-module interactions and between-module links. In this paper, we focus our discussion of the sparse matrix on the results of between-module links, while the results of within-module interactions can be found in the Supplementary Material available online at http://dx.doi.org/10.1155/2015/573956 (Supplementary Data 1).

Low-rank and sparse of matrix structures have been profoundly studied in matrix completion and compressed sensing [10, 11]. The robust principal component analysis (RPCA) [12] proved that the low-rank and the sparse components of a matrix can be exactly recovered if it has a unique and precise “low-rank + sparse” decomposition. RPCA offers a blind separation of low-rank data and sparse noises, which assumed **X** = **L** + **S** (**S** is the sparse noise), and exactly decomposes **X** into **L** and **S** without predefined rank(**L**) and card(**S**). Another successful matrix decomposition method GoDec studied the approximated “low-rank + sparse” decomposition of a matrix **X** by estimating the low-rank part **L** and the sparse part **S** from **X**, allowing noise, that is, **X** = **L** + **S** + **e**, and constrained the rank range of **L** and the cardinality range of *S* [13]. It has been stated that GoDec has outperformed other algorithms before.

In this paper, we modified the GoDec matrix decomposition method and developed “low-rank and sparse decomposition” (LRSDec) to estimate the low-rank part **L** and the sparse part **S** of **X**. LRSDec minimizes the nuclear norm of **L** and predefines the cardinality range of **S**, while considering the additive noise **e**. Different from GoDec, which directly constrains the rank range of **L**, LRSDec minimizes its responding convex polytopes, that is, the nuclear norm of **L**. It has been proven that the nuclear norm outperforms the rank-restricted estimator [14]. Furthermore, if, in presence of missing data, LRSDec could impute the missing entries while decomposing, with no need for data pretreatment, while GoDec could not accomplish decomposition and imputation simultaneously, then we stated the convergence properties of our algorithm and proved that, given the two regularization parameters, the objective value of LRSDec monotonically decreases. By applying both methods to a synthetic dataset, we demonstrated the superiority of LRSDec over GoDec. Finally, we analyzed a genetic interaction dataset (EMAP) using our algorithm and identified many biologically meaningful modules and cross talks between them.

## 2. Model

Let **X** be an *m* × *n* matrix that represents a genetic interaction dataset, where *m* is the number of query genes and *n* is the number of library genes. We propose to decompose **X** as

(1) $$\begin{array}{c} X=L+S+e, \end{array}$$

where **L** ∈ *ℝ* ^*m*×*n*^ denotes the low-rank part and **S** ∈ *ℝ* ^*m*×*n*^ denotes the sparse part, and **e** is the noise. Here, we introduce **L** ∈ *ℝ* ^*m*×*n*^ to account for modules, in which genes are highly correlated. These modules correspond to protein complexes, pathways, and biological pathways, in which genes tend to share similar genetic interaction profiles [15]. **S** ∈ *ℝ* ^*m*×*n*^ is introduced to account for significant *S* scores, which are either gene pairs in the same module that have genetic interactions or cross talks among different functional modules.

Based on the assumptions above, we propose to solve the following optimization problem:

(2) minimize rank(L), minimize  card(S)subject  to  ∑(i,j)Xij−Lij−Sij2⩽δ,

where *δ* ≥ 0 is a regularization parameter that controls the error tolerance, and card(**S**) denote the number of nonzero entries in matrix **S**.

To make the minimization problem tractable, we relax the rank operator on **L** with the nuclear norm, which has been proven to be an effective convex surrogate of the rank operator [14]

(3) minimize  L∗, minimize  card(S)subject  to  ∑(i,j)Xij−Lij−Sij2⩽δ,

where ‖**L**‖_∗_ is the nuclear norm of **L** (‖**L**‖_∗_ = ∑_*i*=1_^*r*^ *σ*_*i*_, where *σ*_1_,…, *σ*_*r*_ are the singular values of **L** and *r* is the rank of **L**).

However, missing data is commonly encountered in EMAP data, confounding techniques such as cluster analysis and matrix factorization. Here, we extend our basic model (3) to handle EMAP data with missing values by imputing missing entries in the matrix simultaneously when estimating low-rank matrix **L** and sparse matrix **S**. Suppose that we only observe a subset of **X**, indexed by *Ω*, and the missing entries are indexed by *Ω* ^⊥^. In order to find a low-rank matrix **L** and a sparse matrix **S** based on the observed data, we propose to solve the following optimization problem:

(4) minimize  L∗, minimize  card(S)subject  to  ∑(i,j)∈ΩXij−Lij−Sij2⩽δ.

## 3. Algorithm

Similar to GoDec, the optimization problem of (3) can be solved by alternatively optimizing the following two subproblems until convergence:

(5a) $$\begin{array}{c} L_{t}=\underset{{\left\|L\right\|}_{*}}{\arg\;\min}{\left\|X-L-S_{t-1}\right\|}_{F}^{2}, \end{array}$$

(5b) $$\begin{array}{c} S_{t}=\underset{\text{card}(S)\leq k}{\arg\;\min}{\left\|X-L_{t}-S\right\|}_{F}^{2}. \end{array}$$

In each iteration, we optimize the objective function by alternatively updating **L** and **S**. Firstly, the subproblem (5a) can be solved by [14]. For fixed **S**, the solution of (5a) is

(6) $$\begin{array}{c} L_{t}=T_{\lambda }\left(X-S_{t-1}\right). \end{array}$$

Here, *λ* ≥ 0 is a regularization parameter controlling the nuclear norm of estimated value **L**_*t*_, where

(7) $$\begin{array}{c} T_{\lambda }\left(W\right)=UD_{\lambda }V^{\prime },\\ \text{with}\;\;D_{\lambda }=\mathrm{diag}\left[{\left(d_{1}-\lambda \right)}_{+},\ldots ,{\left(d_{r}-\lambda \right)}_{+}\right]. \end{array}$$

**U** **D** **V**′ is the* Singular Value Decomposition* (SVD) of **W** and here *t*_+_ = max⁡(*t*, 0). The notation **T**_*λ*_(**W**) refers to* soft-thresholding* [14].

Next, the subproblem (5b) in (3) could be updated via entry-wise hard thresholding of **X** − **L**_*t*_ for fixed **L**_*t*_. Before giving the solution, we define an orthogonal projection operator *𝒫*. Suppose there is a subset of dataset **W**, indexed by *Ω*; then the matrix **W** can be projected onto the linear space of matrices supported by *Ω*:

(8) $$\begin{array}{c} P_{\Omega }{\left(W\right)}_{\left(i,j\right)}=\left\{\begin{array}{cc} W_{ij} & \text{if}\;\left(i,j\right)\in \Omega \\ 0 & \text{if}\;\left(i,j\right)\notin \Omega . \end{array}\right. \end{array}$$

And *𝒫*_*Ω*^⊥^_ is its complementary projection; that is, *𝒫*_*Ω*_(**W**) + *𝒫*_*Ω*^⊥^_(**W**) = **W**.

Then the solution of (5b) could be given as follows:

(9) $$\begin{array}{c} S=P_{\Theta }\left(X-L_{t}\right), \end{array}$$

where *𝒫* is the orthogonal projection operator as defined above, Θ is the nonzero subset of the first *k* largest entries of |(**X** − **L**_*t*_)|. Then, the matrix (**X** − **L**_*t*_) can be projected onto the linear space of matrices supported by Θ:

(10) $$\begin{array}{c} P_{\Theta }{\left(X-L_{t}\right)}_{\left(i,j\right)}=\left\{\begin{array}{cc} {\left(X-L_{t}\right)}_{ij} & \text{if}\;\left(i,j\right)\in \Theta \\ 0 & \text{if}\;\left(i,j\right)\notin \Theta . \end{array}\right. \end{array}$$

So far we have developed the algorithm for solving problem (3). As for problem (4), due to the existence of missing values, we took the optimization on the observed data, *Ω*. We updated **L**_*t*_ and **S**_*t*_ of the following optimization subproblems, respectively:

(11a) $$\begin{array}{c} L_{t}=\underset{{\left\|L\right\|}_{*}}{\arg\;\min}{\left\|P_{\Omega }\left(X-L-S_{t-1}\right)\right\|}_{F}^{2}, \end{array}$$

(11b) $$\begin{array}{c} S_{t}=\underset{\text{card}(S)\leq k}{\arg\;\min}{\left\|P_{\Omega }\left(X-L_{t}-S\right)\right\|}_{F}^{2}. \end{array}$$

The term ‖*𝒫*_*Ω*_(**X** − **L** − **S**)‖_*F*_^2^ is the sum of squared errors on the observed entries indexed by *Ω*.

The subproblem (11a) can be solved by updating **L** with an arbitrary initialization using [14]

(12) $$\begin{array}{c} L_{t}⟵T_{\lambda }\left(P_{\Omega }\left(X-S_{t-1}\right)+P_{\Omega^{⊥}}\left(L\right)\right). \end{array}$$

The solution of subproblem (11b) is

(13) $$\begin{array}{c} S_{t}=P_{\Theta }\left(P_{\Omega }\left(X-L_{t}\right)\right), \end{array}$$

where Θ is the nonzero subset of the first *k* largest entries of |*𝒫*_*Ω*_(**X** − **L**_*t*_)|.

Now we have the following algorithm.

**Algorithm 1 (LRSDec).**

(i) Input: **X** ∈ *ℝ* ^*m*×*n*^. Initialize **S** ← 0.

(ii) Iterate until convergence:

 (a)**L**-step: iteratively update **L** using (12).

 (b)**S**-step: Solve **S** using (13).

(iii) Output: **L**, **S**.

The convergence analysis of our algorithms is provided in the Supplementary Material.

## 4. Parameter Tuning

We have two parameters that need to be tuned in our models: *λ* and *k*. Here, we propose a 10-fold cross validation strategy to select them. The idea is as follows: let *Ω* be the index of observed entries of **X**. We randomly partition *Ω* into 10 equal size subsets and choose training entries *Ω*_1_ and testing entries *Ω*_2_: *Ω*_1_ ∪ *Ω*_2_ = *Ω* and *Ω*_1_∩*Ω*_2_ = *Ø*, |*Ω*_1_ | = 0.9∗ | *Ω* | , |*Ω*_2_ | = 0.1∗ | *Ω*|. We may solve problem (15) on a grid of (*λ*, *k*) values on the training data:

(14a) $$\begin{array}{c} L_{t}=\underset{{\left\|L\right\|}_{*}}{\arg\;\min}{\left\|P_{\Omega_{1}}\left(X-L-S_{t-1}\right)\right\|}_{F}^{2}, \end{array}$$

(14b) $$\begin{array}{c} S_{t}=\underset{\text{card}(S)\leq k}{\arg\;\min}{\left\|P_{\Omega_{1}}\left(X-L_{t}-S\right)\right\|}_{F}^{2}. \end{array}$$

Then we evaluate the prediction error (15) on the testing data:

(15) $$\begin{array}{c} \text{Err}\left(\lambda ,k\right)=\frac{1}{2}{\left\|P_{\Omega_{2}}\left(X-L(\lambda ,k)-S(\lambda ,k)\right)\right\|}_{F}^{2}. \end{array}$$

The cross validation process is repeated for 10 times. Then we can find the optimal parameter (*λ* ^∗^, *k* ^∗^), which minimizes the mean of the prediction error.

## 5. Results

### 5.1. Synthetic Data

We simulated a synthetic data and then applied LRSDec algorithm and GoDec algorithm to it. Specifically, low-rank part, sparse part, and noises are generated as follows.

(i) Low-rank part: the covariance matrix Σ is generated by **H** **H** ^*T*^, where **H** ∈ *ℝ* ^*m*×*K*^ and **H**_*i*,*j*_ ~ *𝒩*(0,1). Here *K* is the number of hidden modules. The random entries **L**_*j*_ are drawn from *𝒩*(0, *τ*Σ). Let **L** = [**L**_1_,…, **L**_*n*_].
(ii) Sparse part: the non-zero entries in sparse matrix are generated from the tail of Gaussian distribution *𝒩*(1,2), whose upper quantile is *α* = 0.01. We randomly selected 70% of them to assign the opposite sign. This is consistent with EMAP datasets, in which negative genetic interactions are much more prevalent than the positive ones.
(iii) **e** = 10^−2^∗**F**, wherein **F** is a standard Gaussian matrix.

A low-rank matrix *L* with rank 25 and sparse matrix with cardinality 250 were generated, respectively. Now we have

(16) $$\begin{array}{c} X=L+S+e. \end{array}$$

**Figure 1:**
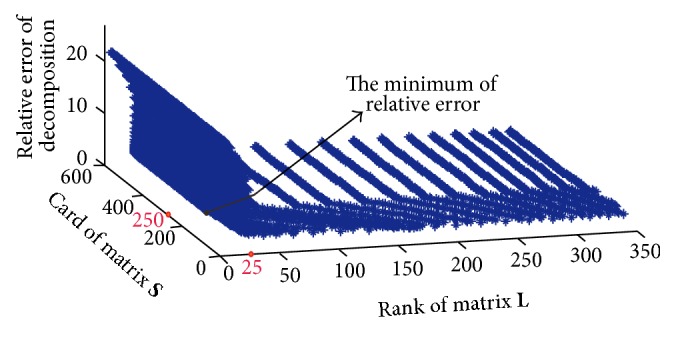
Results of parameter tuning on synthetic data. *x*-axis: different cardinality corresponding to parameter *k*. *y*-axis: different rank corresponding to parameter *λ*. *z*-axis: the relative error: ${\left\|X-\hat{X}\right\|}_{F}^{2}/{\left\|X\right\|}_{F}^{2}$.

The first step is parameter training, and the result is showed in Figure 1. Minimal prediction error was achieved when *λ* = 250 and *k* = 25, which coincides with the rank and cardinality of the synthetic data. This demonstrated the effectiveness of cross validation procedure.

**Figure 2:**
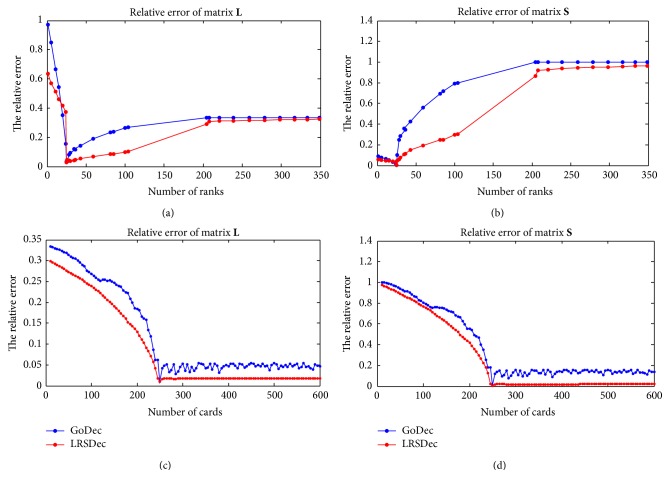
Performances of LRSDec and GoDec in low-rank and sparse decomposition tasks on synthetic data under different parameters. ((a)-(b)) Fixed parameter card, different parameter rank; ((c)-(d)) fixed parameter rank, different parameter card.

Next, we compared the performance of LRSDec algorithm and GoDec algorithm by comparing their prediction error. The relative error is defined as

(17) $$\begin{array}{c} \frac{{\left\|W-\hat{W}\right\|}_{F}^{2}}{{\left\|W\right\|}_{F}^{2}}, \end{array}$$

where **W** is the original matrix and $\hat{W}$ is an estimate/approximation. As both algorithms are influenced seriously by the parameters, we compared the relative error of the two algorithms by given different parameters. To make the comparison simple, we only changed one parameter with another parameter fixed (Figure 2). One can see that both algorithms reach the smallest relative if adopting the correct two parameters, and LRSDec outperforms GoDec. In the Supplementary Material, we also compare the performance of both algorithms under different noise setting, and the trends are the same.

### 5.2. Application to EMAP Data from Yeast

We also applied our method to EMAP data interrogating RNA processing in* S. cerevisiae*, which consists of 552 genes involved in RNA-related processes [9]. This genetic map contains about 152,000 pairwise genetic interaction measurements with about 29% missing entries in data matrix. We applied our method to this EMAP data, denoted as **X**, to obtain two matrices, a low-rank matrix **L** and a sparse matrix **S**. $\hat{X}=L+S$ is the new complete data matrix with imputed missing entries. To exploit the quantitative information from EMAP data, we first subjected the entire low-rank matrix **L** to hierarchical clustering, an approach that groups genes with similar patterns of genetic interactions. It should be noted that using low-rank matrix **L** in cluster improved the performance of hierarchical clustering in detecting genetic interaction modules [16]. According to the clustering result, we reordered rows and columns of matrix $\hat{X}$, so that the protein complexes and biological processes showed in [9] could be found (Figure S1).

**Figure 3:**
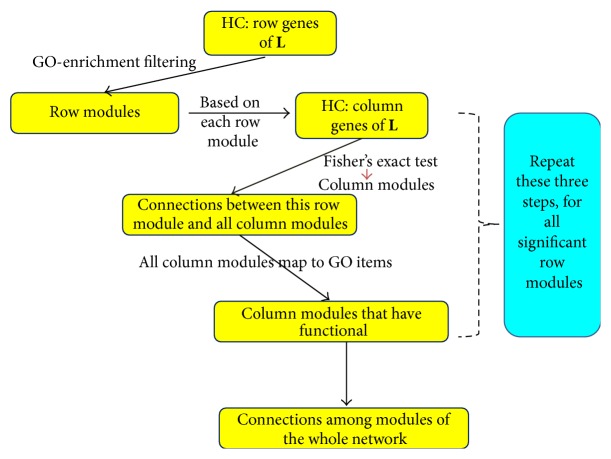
Flowchart of our strategy 1 for detecting modules and cross talks between them in genetic interaction network by low-rank matrix **L**.

**Figure 4:**
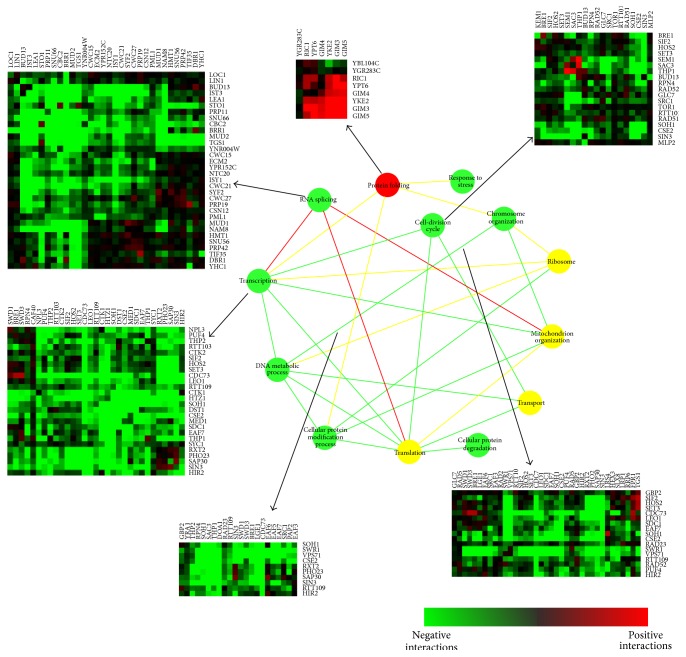
Global view of the genetic cross talks between different RNA-related processes (GO slim items). Green and red represent a statistically significant enrichment of negative (genetic interaction score [*S*]≤−2.5) and positive (genetic interaction score [*S*] > 2.0) interactions, respectively, whereas yellow corresponds to cases where there are roughly equal numbers of positive and negative genetic interactions. Nodes (balls) correspond to distinct functional processes; edges (lines) represent how the processes are genetically connected. The square heat maps represent scores of interactions within one process, and the rectangle heat maps represent scores of interactions between two processes.

**Figure 5:**
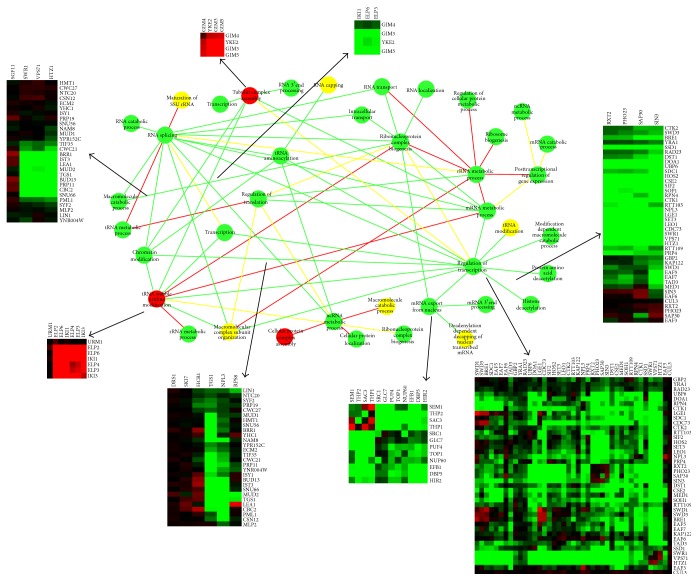
Global view of the genetic cross talks between different RNA-related processes (GO BP FAT). Green and red represent a statistically significant enrichment of negative (genetic interaction score [*S*]≤−2.5) and positive (genetic interaction score [*S*] > 2.0) interactions, respectively, whereas yellow corresponds to cases where there are roughly equal numbers of positive and negative genetic interactions. Nodes (balls) correspond to distinct functional processes; edges (lines) represent how the processes are genetically connected. The square heat maps represent scores of interactions within one process, and the rectangle heat maps represent scores of interactions between two processes.

**Figure 6:**
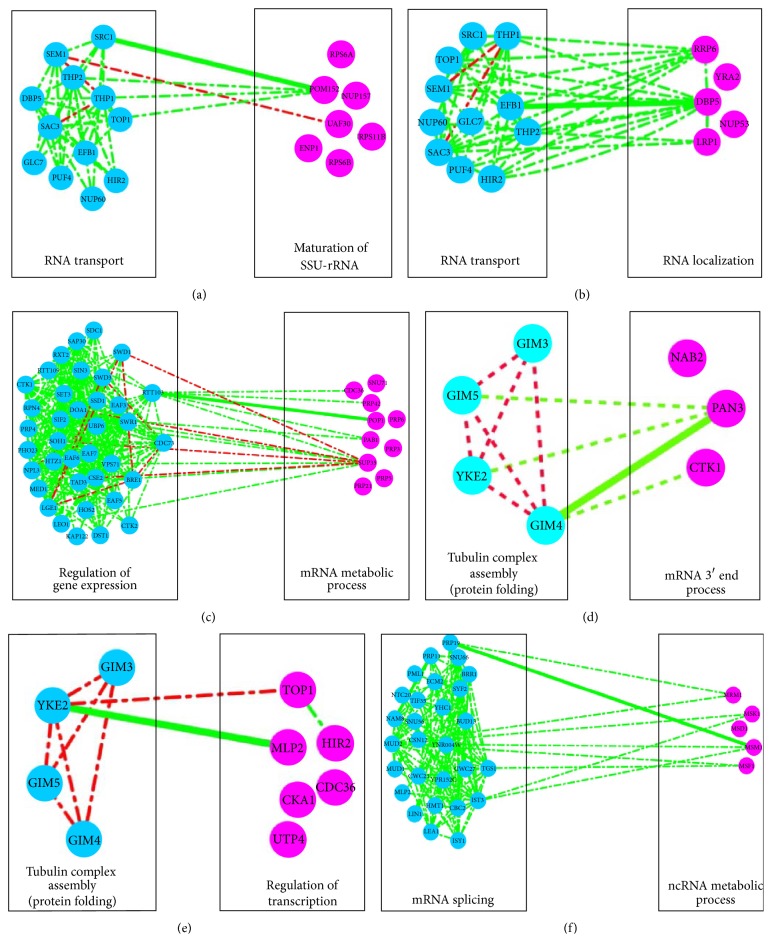
Functional associations between functional modules identified by sparse information. Blue and purplish red represent genes (nodes) involved in different modules. Edges (lines) represent how the processes are genetically connected, where green and red represent a statistically significant enrichment of negative (genetic interaction score [*S*]≤−2.5) and positive (genetic interaction score [*S*] > 2.0) interactions. The emphasized (thicker) lines are the significant *S* scores in sparse matrix.

To help identify more modules of cellular functions and processes and reveal the relationships between them, we further analyzed the matrices **L** and **S**.  Figure 3 is a flowchart of our strategy 1 to detect modules and cross talks between them through low-rank matrix **L**. In this paper, we define module as a cluster from hierarchical clustering (HC) that passes through GO-enriched filtering (Supplementary Section 3). The details are as follows. Firstly, we clustered the row genes of matrix **L** using hierarchical clustering (HC). Here, we adopted the average-linkage hierarchical clustering algorithm in which the distance of gene *A* and gene *B* is defined as 1 − |cor(*A*, *B*)|, where |cor(*A*, *B*)| is the absolute value of the correlation of genetic interaction profile of gene *A* and gene *B*. A cutoff needs to be applied for HC to cut the hierarchical clustering tree. We used the Jaccard index (Supplementary Section 4) to determine how well the predicted gene sets correspond to benchmark (theoretical) gene sets [17]. Here, GO functional gene sets are used as benchmark (theoretical) gene sets. The cutoff at which HC achieved the highest Jaccard index is used to cut the hierarchical tree. We calculated the Jaccard index of every “height” cutoff in hierarchical clustering from 0.2 to 0.95 by 0.05 interval. This step resulted in the best Jaccard index with height = 0.7 and 84 clusters. Now we got the clusters of row genes, in which genes act in a consistent manner across the entire column genes. Then we filtered the clustering results by a hypergeometric test that calculates the significance of enrichment of GO items, and the *p* value was set to 0.01. The clusters enriched in GO functional categories are defined as row modules. Secondly, for each row module, we exploited modules of column genes based on this row gene module in matrix $\hat{X}$ by clustering the column genes of this submatrix of $\hat{X}$. Thirdly, we screened column clusters whose interactions with the row modules are significantly enriched by Fisher's exact test (Table 1, *p* value = 0.05). Here, we defined the positive genetic interactions as those gene pairs with genetic interaction scores *S* ≥ 2.0 and negative as *S* ≤ −2.5 [9]. The reduced gene sets of column genes were defined as column modules (corresponding to the row module). Next we identified the enriched GO functional categories of these column modules by mapping them to GO items (hypergeometric test). Finally, repeating these steps for all row modules, we identified the modules and intermodule genetic cross talk of the whole genetic interaction network (Figures 4–6), where red and green represent a statistically significant enrichment of positive and negative interactions. The cross talks constructed in these figures are the *S* scores among genes in the original matrix. In the following, we will discuss many of the interesting connections that have been reported previously.

The low-rank matrix found more functional modules than the original matrix (Table 2). In Table 2, we cut the dendrogram at different heights and compared the clusters obtained from the low-rank matrix and that of the original matrix. Jaccard index [17] is used to determine how well the predicted clusters recaptured the benchmark gene sets ((a) GO slim and (b) GO BP FAT). The definition of Jaccard index can be found in the Supplementary Material. In the ideal situation where predicted clusters perfectly match the benchmark gene sets, the Jaccard index is 1. The larger the Jaccard index, the better the predictions. The clusters obtained from clustering of the low-rank matrix are more enriched with GO functional categories at varying cutoffs (Table 2).

Figure 4 gives an overview of the relationships among biological processes when GO slim (downloaded from SGD [18], a broad overview of all of the top GO categories) is used as GO items. We found that several sets of genes that have been known to function in the same biochemical processes contain predominantly positive or negative interactions, which was also observed in [9]. For example, genes classed as involved in RNA splicing and transcription are significantly enriched in negative genetic interactions (Figure 4, green nodes). In contrast, the module involved in protein folding has strong positive interactions (red node). In addition, Figure 4 also suggested that not all modules have consistent pattern of interactions (yellow node), which is reasonable in biological processes. Finally, several connections have been previously discussed. For example, there is good evidence for functional interactions between splicing and transcription in [19] and functional interactions between splicing and translation in [20]. Furthermore, [21] reported the cooperative relationship between protein folding and chromosome organization.

Actually, if we classify the GO functional categories to more fine items (GO BP FAT, downloaded from DAVID http://david.abcc.ncifcrf.gov/), we can get a more comprehensive network (Figure 5). Many of the interaction results in Figure 5 have been reported before. For instance, genes involved in tubulin complex assembly process have negative genetic interactions with genes involved in tRNA wobble uridine modification process, supported by SGD, while the negative genetic interactions between RNA splicing process and tRNA metabolic process could also be found. Actually, the genetic interaction between RNA splicing and chromatin modification has been studied in [22]. And the balance of the interactions between the processing of ribonucleoprotein assembly of intronic noncoding RNAs and the splicing process regulating the levels of ncRNA and host mRNA can be found in [23]. Tubulin functionally relating to roles of the elongator complex in tRNA wobble uridine modification is supported by [24]. Moreover, epistasis and chromatin immunoprecipitation experiments indicating that the loss of Rrp6 (regulation of transcription) function is paralleled by the recruitment of Hda1 (histone deacetylase) have been reported by [25]. Finally, cotranscriptional recruitment of the mRNA export factor Yra1 realized by direct interaction with the 3′ end processing factor Pcf11 was in [26].

In an effort to gain insight into the functional organization of RNA-related complexes, we used the GO CC FAT (downloaded from DAVID) as the GO functional categories to create a map that highlights strong genetic trends both within and between these complexes. This result could be found in the Supplementary Material.

Now let us turn to the analysis of the sparse matrix **S**. The sparse matrix gives two distinct measures to exploit genetic information. First, extreme *S* scores indicate cofunctional membership more efficiently [27]. Second, some *S* scores indicate the significant genetic interactions between genes in different gene sets. Following this clue, by analyzing the matrix** S**, we found much evidence of genes involved in the same functional modules and many cross talks between functional modules (Figure 6). Actually, many of them support the network in Figures 4-5. The information of gene pairs with extreme *S* scores and the involved modules could be found in the Supplementary Material (Supplementary Data 2).

Strategy 2 of sparse matrix analysis is similar to strategy 1 (Figure 3). First, for every row module (the same definition as that in Figure 3), cluster the column genes based on their genetic spectrums across genes in this row module. Then select the column gene sets, in which there are genes belonging to nonzero entries of sparse matrix to be defined as column modules. Finally, map these column modules to GO items, identifying their enriched functional categories (hypergeometric test). Similarly, for all row modules, repeat the above steps. Now we got the information of connections between different functional modules (Figure 6).

Several interesting connections become evident when the data is analyzed in this way. For example, there are negative genetic interactions between SRC1 and POM152 [28] and also physical interactions between them [28]. We have found the predominantly negative interactions between RNA transport and maturation of SSU-rRNA (Figure 6(a)). Also we found negative interactions between RNA transport and RNA localization (Figure 6(b)), where the negative interaction between EFB1 and DBP5 revealed in the sparse matrix probably reflects the cross talk between them. Another striking finding is the obviously negative interactions between protein folding and mRNA 3′ end process (Figure 6(d)). PAN3 has negative interactions with GIM4, which has been stated in [9, 29, 30], with GIM5, which has been stated in [29], and with YKE2, which has been stated in [29, 30]. NAB2 was clustered together with PAN3 but showed no obvious genetic interactions with protein folding genes in the original dataset, but in fact it has physical interactions with GIM3, GIM4, and GIM5 [31]. Furthermore, we found cross talk between protein folding and regulation of transcription. Although genes involved in regulation of transcription present low *S* score between each other, they are enriched in the same GO functional item: regulation of transcription (*p* value = 0.017813). More results could be found in Supplementary Material.

## 6. Conclusion

In this paper, we have introduced a method named “LRSDec” to identify gene modules and cross talks between them in the genetic interaction network. LRSDec is based on low-rank approximation with regularization parameters and nearly optimal error bounds. We developed LRSDec to estimate the low-rank part **L** and the sparse part** S** of the original matrix **X**. In the synthetic data, LRSDec performed better than another matrix decomposition algorithm “GoDec,” which has been shown to be other existing decomposition algorithms [13]. Then we applied our algorithm to a genetic interaction dataset to identify modules and cross talks between them. After the decomposition, subsequent analysis revealed many novel and biologically meaningful connections. Moreover, LRSDec could impute missing data while decomposing, which could not be accomplished by other decomposition algorithms. Actually, LRSDec will not be limited by the yeast genetic interaction data. As long as the dataset has internal low-rank structure and some sparse information, we can use the LRSDec algorithm to decompose the data matrix into addition of two matrixes and then analyze them separately. This algorithm could be used widely in the field of genetic interaction data analysis, image processing, and so on. We also had a try on the genetic interaction data of* C. elegans* in the Supplementary Material.

## Supplementary Material

Supplementary data 1 gives the results of sparse S-scores, which are all within modules. Gene pairs with these sparse S-scores are co-functional within gene modules. In this table, column A and B are gene pairs names. Column C are gene pairs belonging cluster numbers, which are from the clustering results. Column D are the gene enriched functional sets, by matching to GO datasets.

## Figures and Tables

**Table 1:** Fisher's exact test table. (Gene set *A*)^⊥^ denotes the complementary set of gene set *A*. #{*AB*} denotes the number of connections between gene set *A* and gene set *B*. #{*AB* ^⊥^} denotes the number of connections between gene set *A* and the complementary set of gene set *B*.

	Gene set *B*	(Gene set *B*)^⊥^
Gene set *A*	#{*AB*}	#{*AB* ^⊥^}
(Gene set *A*)^⊥^	#{*A* ^⊥^ *B*}	#{*A* ^⊥^ *B* ^⊥^}

**Table:** (a) GO slim as benchmark gene set

# Clusters	Low-rank matrix		Original matrix	
	JC-index	# Enriched^@^	JC-index	# Enriched^@^
200	0.063	190	0.022	46
150	0.070	138	0.032	44
100	0.084	90	0.050	50
50	0.088	30	0.067	24

**Table:** (b) GO BP FAT as benchmark gene set

# Clusters	Low-rank matrix		Original matrix	
	JC-index	# Enriched^@^	JC-index	# Enriched^@^
200	0.137	183	0.044	47
150	0.147	142	0.058	50
100	0.155	96	0.091	52
50	0.131	34	0.078	26

@: hypergeometric test applied to test the enrichment of gene sets. Significance level: FDR < = 0.05. # Cluster: the number of clusters to cut off the hierarchical clustering tree. # Enriched: the number of modules predicted by hierarchical clustering enriched in the GO iterms.
