# Supplementary material for: Low-Rank and Sparse Matrix Decomposition for Genetic Interaction Data
Source: Biomed Res Int. 2015 Jul 26;2015:573956. doi: 10.1155/2015/573956 (PMC4529927; doi:10.1155/2015/573956)
Supplement: Supplementary file 1 — Supplementary data 1 gives the results of sparse S-scores, which are all within modules. Gene pairs with these sparse S-scores are co-functional within gene modules. In this table, column A and B are gene pairs names. Column C are gene pairs belonging cluster numbers, which are from the clustering results. Column D are the gene enriched functional sets, by matching to GO datasets. [file 573956.f1.pdf]

# Supplementary Document of "Low Rank and Sparse Matrix Decomposition for Genetic Interaction Data"

Yishu Wang, Dejie Yang, Minghua Deng

January 8, 2015

## 1 Algorithm convergence analysis

In this section we study the convergence properties of Algorithm LRSDec. Firstly, we define the objective value (decomposition error) is $\|\mathbf{X} - \mathbf{L} - \mathbf{S}\|_F^2$. We have the following lemma about the convergence of the objective value $\|\mathbf{X} - \mathbf{L} - \mathbf{S}\|_F^2$ in (7).

***Lemma 2.*** *(Convergence of objective value)* The alternative optimization (7) produces a sequence of $\|\mathbf{X} - \mathbf{L} - \mathbf{S}\|_F^2$ that converges to a local minimum.

*Proof.* Let the objective value $\|\mathbf{X} - \mathbf{L} - \mathbf{S}\|_F^2$ after solving the two subproblems in (7) be $E_t^1$ and $E_t^2$, respectively, in the $t^{th}$ iteration. On one hand, we have:

$$E_t^1 = \|\mathbf{X} - \mathbf{L}_t - \mathbf{S}_{t-1}\|_F^2, E_t^2 = \|\mathbf{X} - \mathbf{L}_t - \mathbf{S}_t\|_F^2 \tag{1}$$

The global optimality of $\mathbf{S}_t$ yields $E_t^1 \geq E_t^2$. On the other hand,

$$E_t^2 = \|\mathbf{X} - \mathbf{L}_t - \mathbf{S}_{t-1}\|_F^2, E_{t+1}^1 = \|\mathbf{X} - \mathbf{L}_{t+1} - \mathbf{S}_t\|_F^2 \tag{2}$$

The global optimality of $L_{t+1}$ yields $E_t^2 \geq E_{t+1}^1$. Therefore, the objective values (decomposition errors) $\|\mathbf{X} - \mathbf{L} - \mathbf{S}\|_F^2$ keep decreasing throughout LRSDec (7):

$$E_1^1 \geq E_1^2 \geq E_2^1 \geq ... \geq E_t^1 \geq E_t^2 \geq E_{t+1}^1 \geq ... \tag{3}$$

Since the objective of (7) is monotonically decreasing and the constrains are satisfied all the time, the LRSDec algorithm produces a sequence of objective values that converge to a local minimum.

In Section 1.1, we will show that the sequence $\mathbf{L}_t, \mathbf{S}_t$ generated via LRSDec converges asymptoticcally.

## 1.1 Asymptotic Convergence

***Lemma 3.*** The nuclear norm shrinkage operator $\mathbf{T}_\lambda(\cdot)$, defined in *Lemma* 1 and card shrinkage operator $\Lambda_k(\cdot)$, defined in (13), satisfies the following for any $\mathbf{W}_1, \mathbf{W}_2$ (with matching dimensions)

$$\|\mathbf{T}_\lambda(\mathbf{W}_1) - \mathbf{T}_\lambda(\mathbf{W}_2)\|_F^2 \leq \|\mathbf{W}_1 - \mathbf{W}_2\|_F^2$$

$$\|\Lambda_k(\mathbf{W}_1) - \Lambda_k(\mathbf{W}_2))\|_F^2 \leq \|\mathbf{W}_1 - \mathbf{W}_2\|_F^2$$

In particular this implies that $\mathbf{T}_\lambda(\mathbf{W})$ and $\Lambda_k(\mathbf{W})$ are continuous map in $\mathbf{W}$.

*Proof.* The continuity of nuclear norm shrinkage operator $\mathbf{T}_\lambda(\cdot)$ has been proved in [2]. We give the proof of card shrinkage operator $\Lambda_k(\cdot)$.

$$\mathbf{W}_1 = \mathcal{P}_\Theta(\mathbf{W}_1) + \mathcal{P}_{\Theta^\perp}(\mathbf{W}_1), \mathbf{W}_2 = \mathcal{P}_\Theta(\mathbf{W}_2) + \mathcal{P}_{\Theta^\perp}(\mathbf{W}_2) \qquad \Theta \cap \Theta^\perp = \oslash$$

$$\begin{aligned}\|\mathbf{W}_1 - \mathbf{W}_2\|_F^2 &= \|\mathcal{P}_\Theta(\mathbf{W}_1) - \mathcal{P}_\Theta(\mathbf{W}_2) + \mathcal{P}_{\Theta^\perp}(\mathbf{W}_1) - \mathcal{P}_{\Theta^\perp}(\mathbf{W}_2\|_F^2 \\ &= \|\mathcal{P}_\Theta(\mathbf{W}_1) - \mathcal{P}_\Theta(\mathbf{W}_2)\|_F^2 + \|\mathcal{P}_{\Theta^\perp}(\mathbf{W}_1) - \mathcal{P}_{\Theta^\perp}(\mathbf{W}_2\|_F^2 \\ &= \|\Lambda_k(\mathbf{W}_1) - \Lambda_k(\mathbf{W}_2))\|_F^2 + \|\mathcal{P}_{\Theta^\perp}(\mathbf{W}_1) - \mathcal{P}_{\Theta^\perp}(\mathbf{W}_2\|_F^2 \\ &\geq \|\Lambda_k(\mathbf{W}_1) - \Lambda_k(\mathbf{W}_2))\|_F^2\end{aligned}$$

***Lemma 4.*** The successive differences $\|\mathbf{L}_t - \mathbf{L}_{t-1}\|_F^2, \|\mathbf{S}_t - \mathbf{S}_{t-1}\|_F^2$ of the sequence $\mathbf{L}_t, \mathbf{S}_t$ are monotone decreasing:

$$\|\mathbf{L}_{t+1} - \mathbf{L}_t\|_F^2 \leq \|\mathbf{L}_t - \mathbf{L}_{t-1}\|_F^2 \quad \forall t.$$

$$\|\mathbf{S}_{t+1} - \mathbf{S}_t\|_F^2 \leq \|\mathbf{S}_t - \mathbf{S}_{t-1}\|_F^2 \quad \forall t.$$

*Proof.*

$$\begin{aligned}\|\mathbf{L}_{t+1} - \mathbf{L}_t\|_F^2 &= \|\mathbf{T}_\lambda(\mathbf{X} - \mathbf{S}_t) - \mathbf{T}_\lambda(\mathbf{X} - \mathbf{S}_{t-1})\|_F^2 \\ (\textit{by Lemma } 3) &\leq \|(\mathbf{X} - \mathbf{S}_t) - (\mathbf{X} - \mathbf{S}_{t-1})\|_F^2 \\ &= \|\mathbf{S}_{t-1} - \mathbf{S}_t\|_F^2 \\ &= \|\Lambda_k(\mathbf{X} - \mathbf{L}_{t-1}) - \Lambda_k(\mathbf{X} - \mathbf{L}_t)\|_F^2 \\ (\textit{by Lemma } 3) &\leq \|\mathbf{L}_t - \mathbf{L}_{t-1}\|_F^2\end{aligned}$$

In the same way for sequence $\mathbf{S}_t$:

$$\begin{aligned}\|\mathbf{S}_{t+1} - \mathbf{S}_t\|_F^2 &= \|\Lambda_k(\mathbf{X} - \mathbf{L}_{t+1}) - \Lambda_k(\mathbf{X} - \mathbf{L}_t)\|_F^2 \\ &\leq \|\mathbf{L}_t - \mathbf{L}_{t+1}\|_F^2 \\ &= \|\mathbf{T}_\lambda(\mathbf{X} - \mathbf{S}_{t-1}) - \mathbf{T}_\lambda(\mathbf{X} - \mathbf{S}_t)\|_F^2 \\ &\leq \|\mathbf{S}_t - \mathbf{S}_{t-1}\|_F^2\end{aligned}$$

The above implies that sequence $\|\mathbf{L}_t - \mathbf{L}_{t-1}\|_F^2$ and $\|\mathbf{S}_t - \mathbf{S}_{t-1}\|_F^2$ converge (since they are decreasing and bounded below). This implies that:

$$\|\mathbf{L}_{t+1} - \mathbf{L}_t\|_F^2 - \|\mathbf{L}_t - \mathbf{L}_{t-1}\|_F^2 \to 0 \quad as \;\; t \to \infty$$

$$\|\mathbf{S}_{t+1} - \mathbf{S}_t\|_F^2 - \|\mathbf{S}_t - \mathbf{S}_{t-1}\|_F^2 \to 0 \quad as \;\; t \to \infty$$

So there exist constants $\alpha_1 \geq 0, \alpha_2 \geq 0$

$$\|\mathbf{L}_{t+1} - \mathbf{L}_t\|_F^2 \to \alpha_1 \quad as \;\; t \to \infty$$

$$\|\mathbf{S}_{t+1} - \mathbf{S}_t\|_F^2 \to \alpha_2 \quad as \;\; t \to \infty$$

Actually, since LRSDec can be written as the form of alternating projections on two manifolds. According to [1], $\mathbf{L}_t$ converges asymptotically to some point $\mathbf{L}_*$, $\mathbf{S}_t$ converges linearly to some point $\mathbf{S}_*$, for some constant $\alpha$, exists $\beta$:

$$\|\mathbf{L}_t - \mathbf{L}_*\|_F^2 \leq \alpha_1 \beta_1^t$$

$$\|\mathbf{S}_t - \mathbf{S}_*\|_F^2 \leq \alpha_2 \beta_2^t$$

# 2 Figure S1. Hierarchical clustergram of all 552 genes in Section 6.2 with imputing missing values

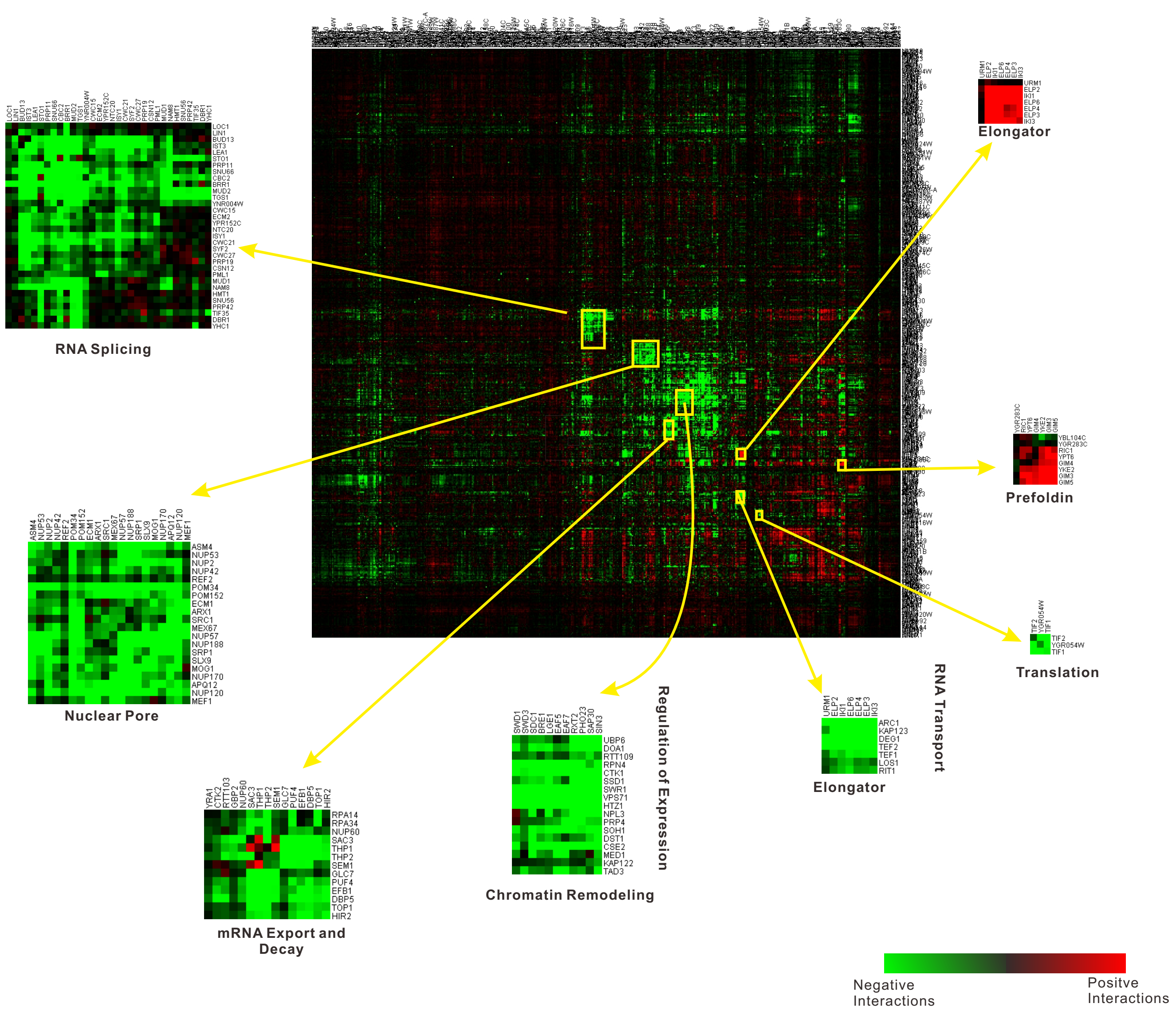


Figure 1: Red and green represent positive and negative genetic interactions, respectively, grey entries in the original figure in ([4]) have been imputed, whose the clustering results could be found more clearly here.

# 3 Calculation of $p$-value for a gene set

Let N be the total number of genes and M be the number of genes related to a functional category from the total genes. Suppose now we have a gene set with $N_1$ genes. Among these $N_1$ genes there are $M_1$ genes related to GO functional category. The $p$-value of this gene set is given below:

$$p(N, M, N_1, M_1) = \sum_{i=M_1}^{N_1} \frac{\begin{pmatrix} M \\ i \end{pmatrix} \begin{pmatrix} N-M \\ N-i \end{pmatrix}}{\begin{pmatrix} N \\ N_1 \end{pmatrix}}$$

The $p$-value are adjusted using Bonferroni correction.

# 4 Jaccard index: evaluation measure of the predicted modules

The Jaccard index [3] between two sets $M_i$ and $B_j$ is defined as:

$$\frac{\sharp\{M_i \cap B_j\}}{\sharp\{M_i \cup B_j\}} \tag{4}$$

where $\sharp\{A\}$ denotes the number of set A

For module $M_i$, the Jaccard index between $M_i$ and each gene set $B_j$ in the benchmark is computed, and the Jaccard index of $M_i$ and the benchmark gene sets is defined as the maximum of Jaccard index between $M_i$ and any gene set in the benchmark:

$$Jaccard\ Index(M_i, B) = max_j\{JaccardIndex(M_i, B_j)\} \tag{5}$$

Thus, the average Jaccard index of the predicted modules and the benchmark gene sets can be computed as:

$$Jaccard\ Index(M, B) = \frac{\sum_{i \in 1,\ldots k} Jaccard\ Index(M_i, B)}{k} \tag{6}$$

## 5 Results in Strategy 2

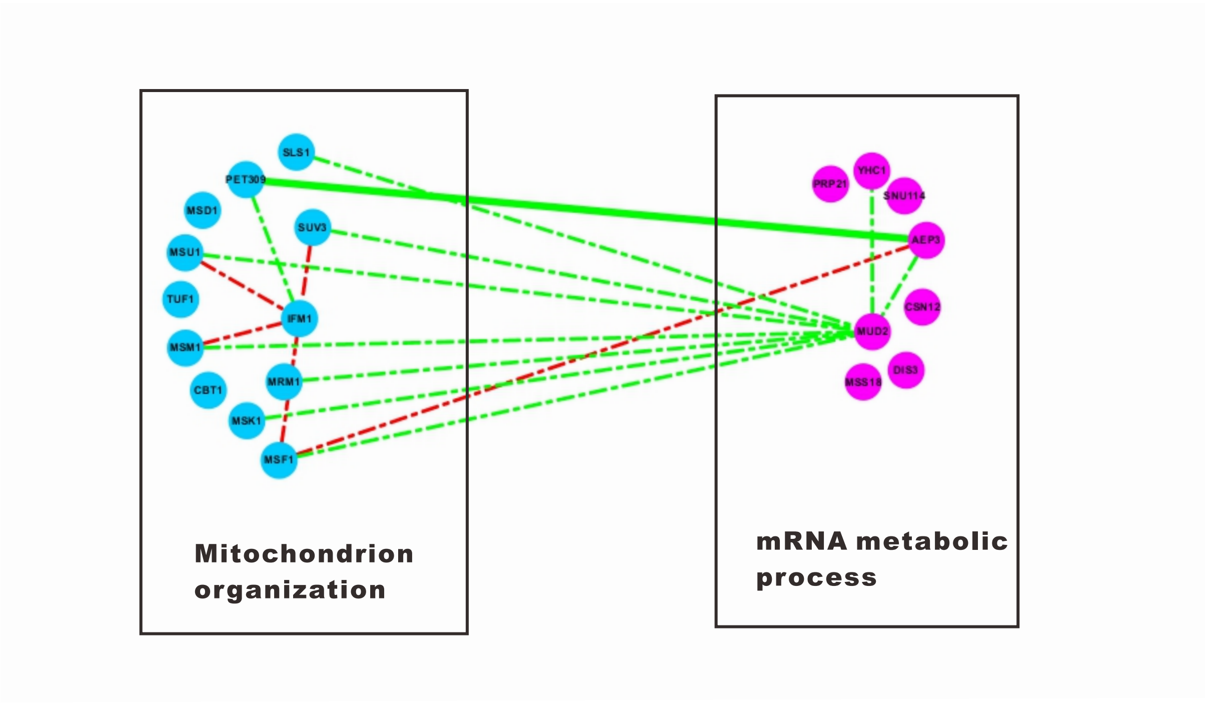


Figure 2: Global

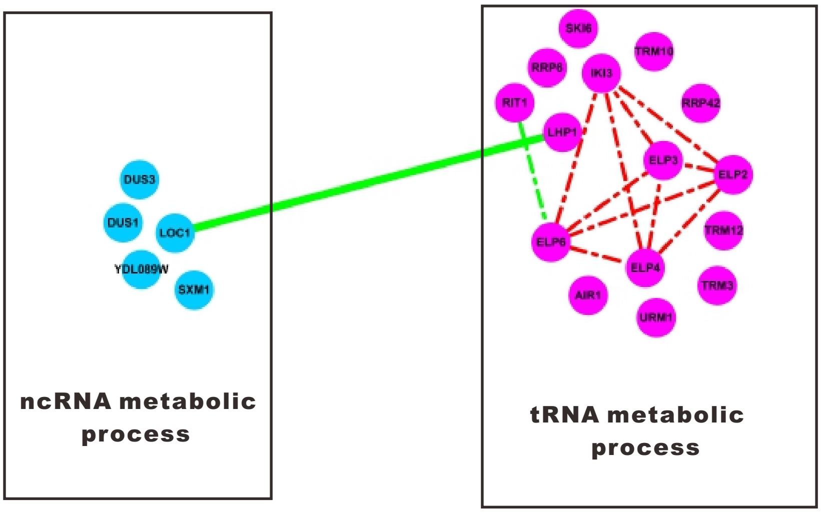


Figure 3: Global

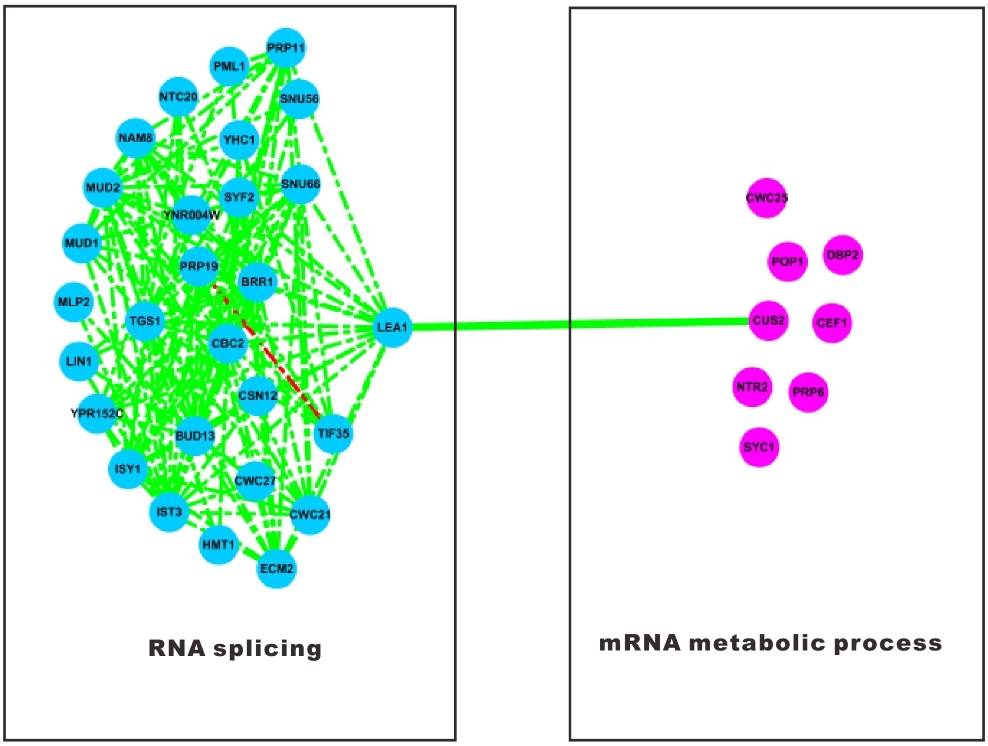


Figure 4: Global

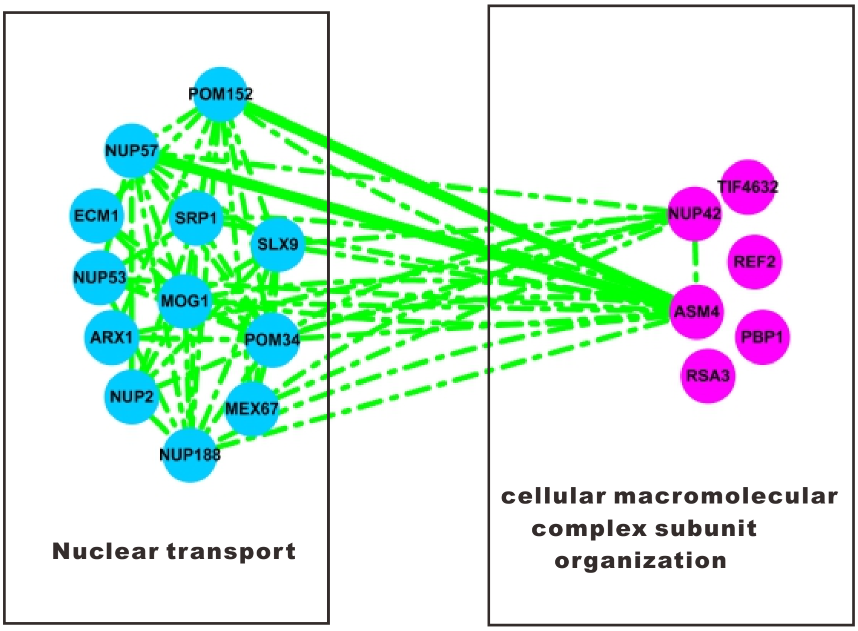


Figure 5: Global

# 6 Results in Synthetic data

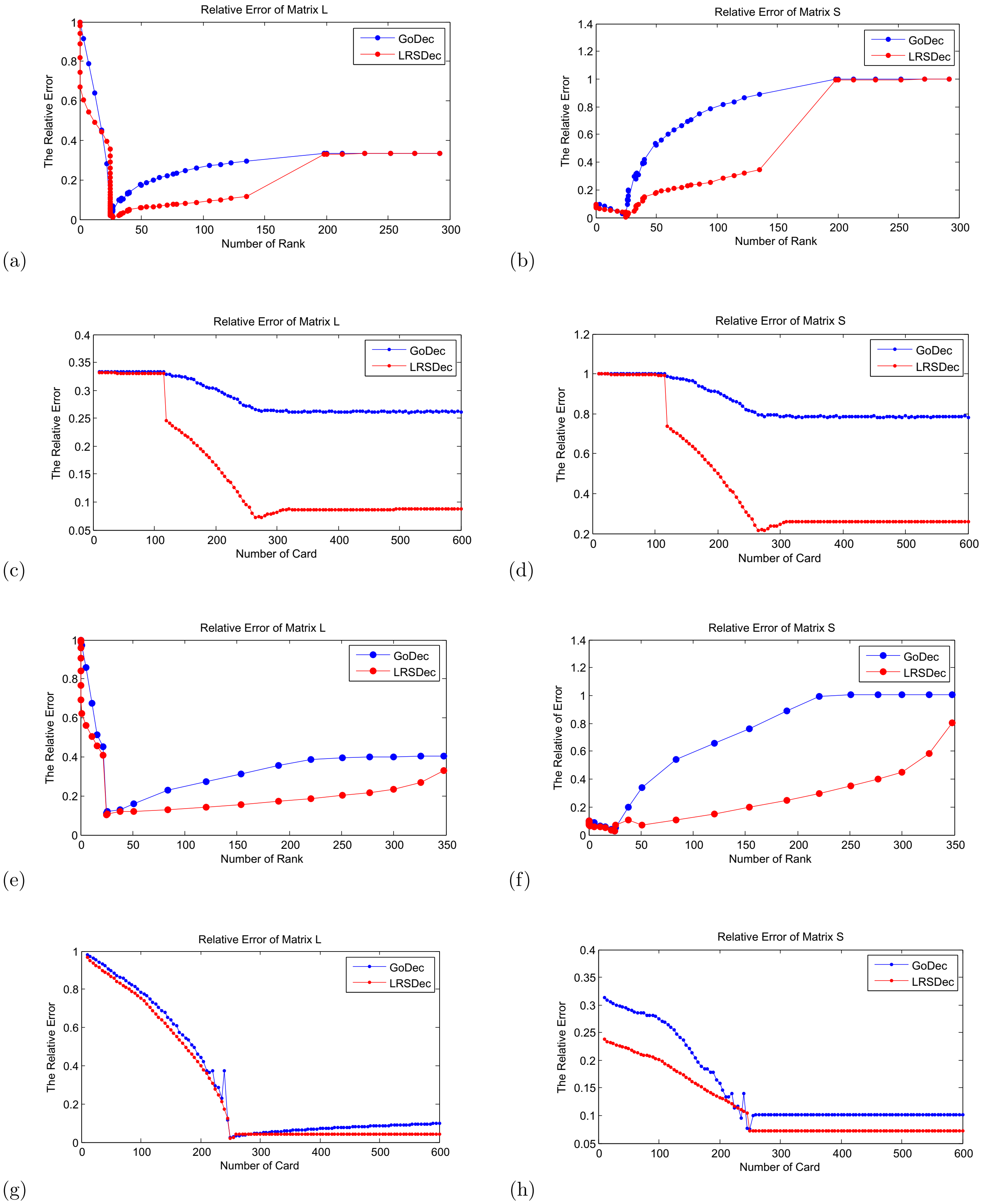


Figure 6: Performances of LRSDec and GoDec in Low-Rank and Sparse decomposition tasks on synthetic data under different paramenters. (a)-(d): nosise $\mathbf{e} = 10^{-3} * \mathbf{F}$, specially, (a)-(b): fixed parameter card, different parameter rank; (c)-(d): fixed parameter rank, different parameter card. And (e)-(h): nosise $\mathbf{e} = 10^{-1} * \mathbf{F}$, specially, (e)-(f): fixed parameter card, different parameter rank; (g)-(h): fixed parameter rank, different parameter card.

# References

[1] Adrian S Lewis and Jérôme Malick. Alternating projections on manifolds. *Mathematics of Operations Research*, 33(1):216–234, 2008.

[2] Rahul Mazumder, Trevor Hastie, and Robert Tibshirani. Spectral regularization algorithms for learning large incomplete matrices. *The Journal of Machine Learning Research*, 99:2287–2322, 2010.

[3] Jimin Song and Mona Singh. How and when should interactome-derived clusters be used to predict functional modules and protein function? *Bioinformatics*, 25(23):3143–3150, 2009.

[4] Gwendolyn M Wilmes, Megan Bergkessel, Sourav Bandyopadhyay, Michael Shales, Hannes Braberg, Gerard Cagney, Sean R Collins, Gregg B Whitworth, Tracy L Kress, Jonathan S Weissman, et al. A genetic interaction map of rna-processing factors reveals links between sem1/dss1-containing complexes and mrna export and splicing. *Molecular cell*, 32(5):735–746, 2008.
