# Supplementary material for: Low-Rank and Sparse Matrix Decomposition for Genetic Interaction Data
Source: Biomed Res Int. 2015 Jul 26;2015:573956. doi: 10.1155/2015/573956 (PMC4529927; doi:10.1155/2015/573956)
Supplement: Supplementary file 2 [file 573956.f2.zip › 573956.f2/Supplementary materials/Low-Sparse_planlatex-Supplementary.pdf]

Supplementary Document of "Low Rank and Sparse Matrix Decomposition for Genetic Interaction Data"

Yishu Wang, Dejie Yang, Minghua Deng

March 15, 2015

1 Algorithm convergence analysis

In this section we study the convergence properties of Algorithm LRSDec. Firstly, we define the objective value (decomposition error) is $\|\mathbf{X} - \mathbf{L} - \mathbf{S}\|_F^2$. We have the following lemma about the convergence of the objective value $\|\mathbf{X} - \mathbf{L} - \mathbf{S}\|_F^2$ in (7).

Lemma 2. *(Convergence of objective value)* The alternative optimization (7) produces a sequence of $\|\mathbf{X} - \mathbf{L} - \mathbf{S}\|_F^2$ that converges to a local minimum.

Proof. Let the objective value $\|\mathbf{X} - \mathbf{L} - \mathbf{S}\|_F^2$ after solving the two subproblems in (7) be E_t^1 and E_t^2, respectively, in the tth iteration. On one hand, we have:

$$E_t^1 = \|\mathbf{X} - \mathbf{L}_t - \mathbf{S}_{t-1}\|_F^2, E_t^2 = \|\mathbf{X} - \mathbf{L}_t - \mathbf{S}_t\|_F^2 \tag{1}$$

The global optimality of $\mathbf{S}_t$ yields $E_t^1 \geq E_t^2$. On the other hand,

$$E_t^2 = \|\mathbf{X} - \mathbf{L}_t - \mathbf{S}_{t-1}\|_F^2, E_{t+1}^1 = \|\mathbf{X} - \mathbf{L}_{t+1} - \mathbf{S}_t\|_F^2 \tag{2}$$

The global optimality of L_{t+1} yields $E_t^2 \geq E_{t+1}^1$. Therefore, the objective values (decomposition errors) $\|\mathbf{X} - \mathbf{L} - \mathbf{S}\|_F^2$ keep decreasing throughout LRSDec (7):

$$E_1^1 \geq E_1^2 \geq E_2^1 \geq ... \geq E_t^1 \geq E_t^2 \geq E_{t+1}^1 \geq ... \tag{3}$$

Since the objective of (7) is monotonically decreasing and the constrains are satisfied all the time, the LRSDec algorithm produces a sequence of objective values that converge to a local minimum.

In Section 1.1, we will show that the sequence $\mathbf{L}_t, \mathbf{S}_t$ generated via LRSDec converges asymptotically.

1.1 Asymptotic Convergence

Lemma 3. The nuclear norm shrinkage operator $\mathbf{T}_\lambda(\cdot)$, defined in *Lemma* 1 and card shrinkage operator $\Lambda_k(\cdot)$, defined in (13), satisfies the following for any $\mathbf{W}_1, \mathbf{W}_2$ (with matching dimensions)

$$\|\mathbf{T}_\lambda(\mathbf{W}_1) - \mathbf{T}_\lambda(\mathbf{W}_2)\|_F^2 \leq \|\mathbf{W}_1 - \mathbf{W}_2\|_F^2$$

$$\|\Lambda_k(\mathbf{W}_1) - \Lambda_k(\mathbf{W}_2))\|_F^2 \leq \|\mathbf{W}_1 - \mathbf{W}_2\|_F^2$$

In particular this implies that $\mathbf{T}_\lambda(\mathbf{W})$ and $\Lambda_k(\mathbf{W})$ are continuous map in $\mathbf{W}$.

Proof. The continuity of nuclear norm shrinkage operator $\mathbf{T}_\lambda(\cdot)$ has been proved in [3]. We give the proof of card shrinkage operator $\Lambda_k(\cdot)$.

$$\mathbf{W}_1 = \mathcal{P}_\Theta(\mathbf{W}_1) + \mathcal{P}_{\Theta^\perp}(\mathbf{W}_1), \mathbf{W}_2 = \mathcal{P}_\Theta(\mathbf{W}_2) + \mathcal{P}_{\Theta^\perp}(\mathbf{W}_2) \qquad \Theta \cap \Theta^\perp = \oslash$$

$$\begin{aligned}\|\mathbf{W}_1 - \mathbf{W}_2\|_F^2 &= \|\mathcal{P}_\Theta(\mathbf{W}_1) - \mathcal{P}_\Theta(\mathbf{W}_2) + \mathcal{P}_{\Theta^\perp}(\mathbf{W}_1) - \mathcal{P}_{\Theta^\perp}(\mathbf{W}_2\|_F^2 \\ &= \|\mathcal{P}_\Theta(\mathbf{W}_1) - \mathcal{P}_\Theta(\mathbf{W}_2)\|_F^2 + \|\mathcal{P}_{\Theta^\perp}(\mathbf{W}_1) - \mathcal{P}_{\Theta^\perp}(\mathbf{W}_2\|_F^2 \\ &= \|\Lambda_k(\mathbf{W}_1) - \Lambda_k(\mathbf{W}_2))\|_F^2 + \|\mathcal{P}_{\Theta^\perp}(\mathbf{W}_1) - \mathcal{P}_{\Theta^\perp}(\mathbf{W}_2\|_F^2 \\ &\geq \|\Lambda_k(\mathbf{W}_1) - \Lambda_k(\mathbf{W}_2))\|_F^2\end{aligned}$$

Lemma 4. The successive differences $\|\mathbf{L}_t - \mathbf{L}_{t-1}\|_F^2, \|\mathbf{S}_t - \mathbf{S}_{t-1}\|_F^2$ of the sequence $\mathbf{L}_t, \mathbf{S}_t$ are monotone decreasing:

$$\|\mathbf{L}_{t+1} - \mathbf{L}_t\|_F^2 \leq \|\mathbf{L}_t - \mathbf{L}_{t-1}\|_F^2 \quad \forall t.$$

$$\|\mathbf{S}_{t+1} - \mathbf{S}_t\|_F^2 \leq \|\mathbf{S}_t - \mathbf{S}_{t-1}\|_F^2 \quad \forall t.$$

Proof.

$$\begin{aligned}\|\mathbf{L}_{t+1} - \mathbf{L}_t\|_F^2 &= \|\mathbf{T}_\lambda(\mathbf{X} - \mathbf{S}_t) - \mathbf{T}_\lambda(\mathbf{X} - \mathbf{S}_{t-1})\|_F^2 \\ (\textit{by Lemma } 3) &\leq \|(\mathbf{X} - \mathbf{S}_t) - (\mathbf{X} - \mathbf{S}_{t-1})\|_F^2 \\ &= \|\mathbf{S}_{t-1} - \mathbf{S}_t\|_F^2 \\ &= \|\Lambda_k(\mathbf{X} - \mathbf{L}_{t-1}) - \Lambda_k(\mathbf{X} - \mathbf{L}_t)\|_F^2 \\ (\textit{by Lemma } 3) &\leq \|\mathbf{L}_t - \mathbf{L}_{t-1}\|_F^2\end{aligned}$$

In the same way for sequence $\mathbf{S}_t$:

$$\begin{aligned}\|\mathbf{S}_{t+1} - \mathbf{S}_t\|_F^2 &= \|\Lambda_k(\mathbf{X} - \mathbf{L}_{t+1}) - \Lambda_k(\mathbf{X} - \mathbf{L}_t)\|_F^2 \\ &\leq \|\mathbf{L}_t - \mathbf{L}_{t+1}\|_F^2 \\ &= \|\mathbf{T}_\lambda(\mathbf{X} - \mathbf{S}_{t-1}) - \mathbf{T}_\lambda(\mathbf{X} - \mathbf{S}_t)\|_F^2 \\ &\leq \|\mathbf{S}_t - \mathbf{S}_{t-1}\|_F^2\end{aligned}$$

The above implies that sequence $\|\mathbf{L}_t - \mathbf{L}_{t-1}\|_F^2$ and $\|\mathbf{S}_t - \mathbf{S}_{t-1}\|_F^2$ converge (since they are decreasing and bounded below). This implies that:

$$\|\mathbf{L}_{t+1} - \mathbf{L}_t\|_F^2 - \|\mathbf{L}_t - \mathbf{L}_{t-1}\|_F^2 \to 0 \quad as \;\; t \to \infty$$

$$\|\mathbf{S}_{t+1} - \mathbf{S}_t\|_F^2 - \|\mathbf{S}_t - \mathbf{S}_{t-1}\|_F^2 \to 0 \quad as \;\; t \to \infty$$

So there exist constants $\alpha_1 \geq 0, \alpha_2 \geq 0$

$$\|\mathbf{L}_{t+1} - \mathbf{L}_t\|_F^2 \to \alpha_1 \quad as \;\; t \to \infty$$

$$\|\mathbf{S}_{t+1} - \mathbf{S}_t\|_F^2 \to \alpha_2 \quad as \;\; t \to \infty$$

Actually, since LRSDec can be written as the form of alternating projections on two manifolds. According to [2], $\mathbf{L}_t$ converges asymptotically to some point $\mathbf{L}_*$, $\mathbf{S}_t$ converges linearly to some point $\mathbf{S}_*$, for some constant α, exists β:

$$\|\mathbf{L}_t - \mathbf{L}_*\|_F^2 \leq \alpha_1 \beta_1^t$$

$$\|\mathbf{S}_t - \mathbf{S}_*\|_F^2 \leq \alpha_2 \beta_2^t$$

2 Figure S1. Hierarchical clustergram of all 552 genes in Section 6.2 with imputing missing values

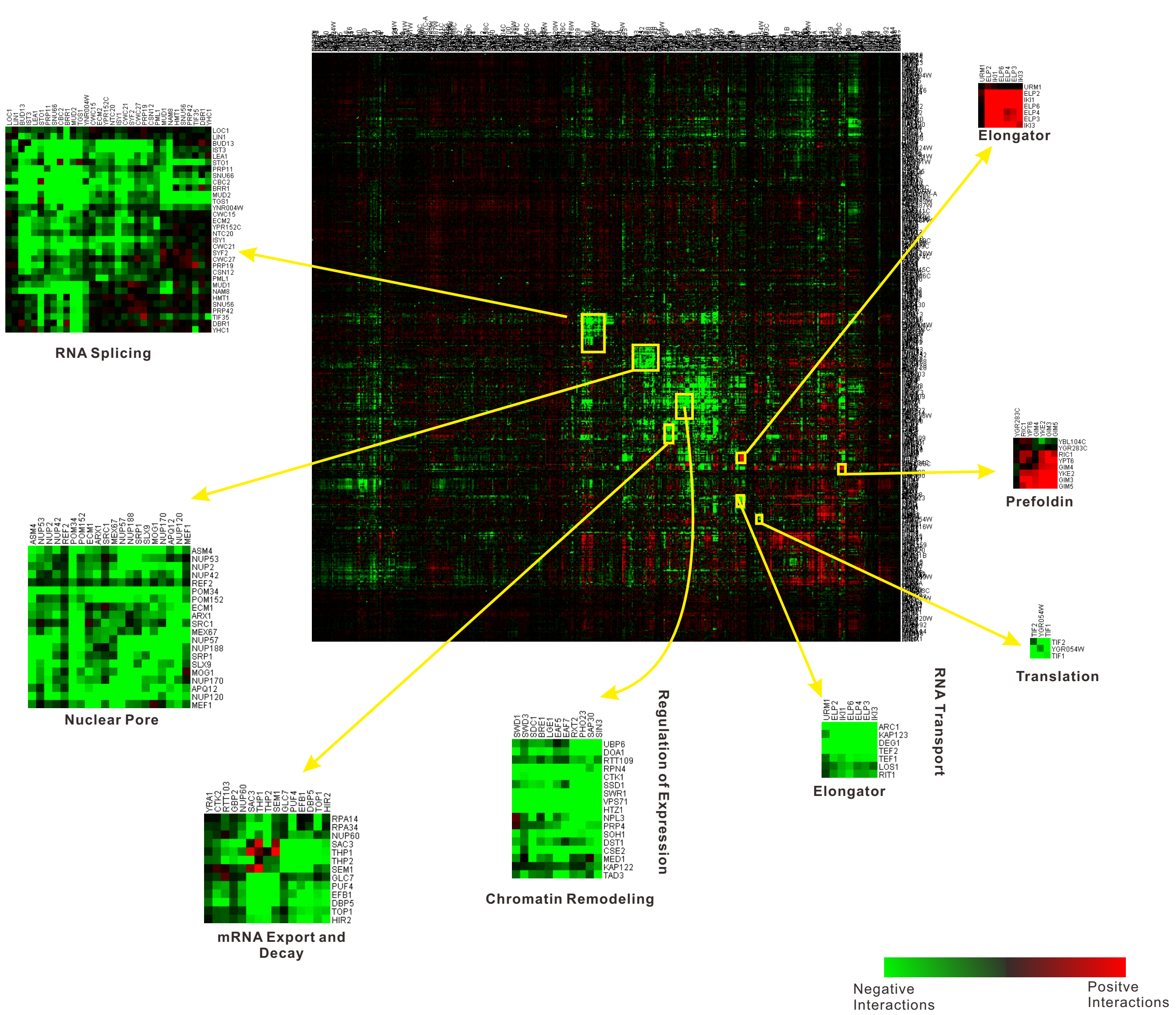

Figure 1: Red and green represent positive and negative genetic interactions, respectively, grey entries in the original figure in ([5]) have been imputed, whose the clustering results could be found more clearly here.

3 Calculation of p-value for a gene set

Let N be the total number of genes and M be the number of genes related to a functional category from the total genes. Suppose now we have a gene set with N_1 genes. Among these N_1 genes there are M_1 genes related to GO functional category. The p-value of this gene set is given below:

$$p(N, M, N_1, M_1) = \sum_{i=M_1}^{N_1} \frac{\begin{pmatrix} M \\ i \end{pmatrix} \begin{pmatrix} N-M \\ N-i \end{pmatrix}}{\begin{pmatrix} N \\ N_1 \end{pmatrix}}$$

The p-value are adjusted using Bonferroni correction.

4 Jaccard index: evaluation measure of the predicted modules

The Jaccard index [4] between two sets M_i and B_j is defined as:

$$\frac{\sharp\{M_i \cap B_j\}}{\sharp\{M_i \cup B_j\}} \tag{4}$$

where $\sharp$\{A\} denotes the number of set A

For module M_i, the Jaccard index between M_i and each gene set B_j in the benchmark is computed, and the Jaccard index of M_i and the benchmark gene sets is defined as the maximum of Jaccard index between M_i and any gene set in the benchmark:

$$Jaccard\ Index(M_i, B) = max_j\{JaccardIndex(M_i, B_j)\} \tag{5}$$

Thus, the average Jaccard index of the predicted modules and the benchmark gene sets can be computed as:

$$Jaccard\ Index(M, B) = \frac{\sum_{i \in 1, \ldots k} Jaccard\ Index(M_i, B)}{k} \tag{6}$$

5 Global view of the genetic crosstalk between different RNA-related complexes

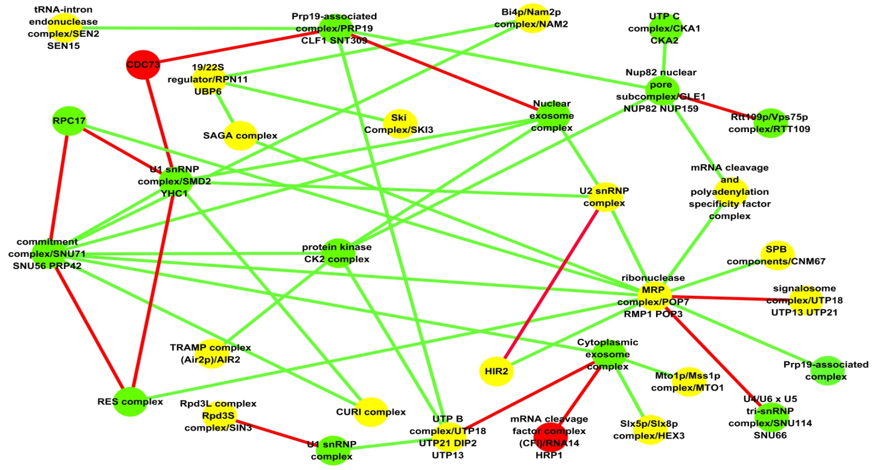

Figure 2: Global view of the genetic crosstalk between different RNA-related complexes (GO CC FAT). Green and red represent a statistically significant enrichment of negative (genetic interaction score $[S] \leq -2.5$)and positive (genetic interaction score $[S] > 2.0$) interactions, respectively, whereas yellow corresponds to cases where there are roughly equal numbers of positive and negative genetic interactions. Nodes (balls) correspond to distinct protein complex, edges (lines) represent how the complexes are genetically connected.

6 Results in Strategy 2

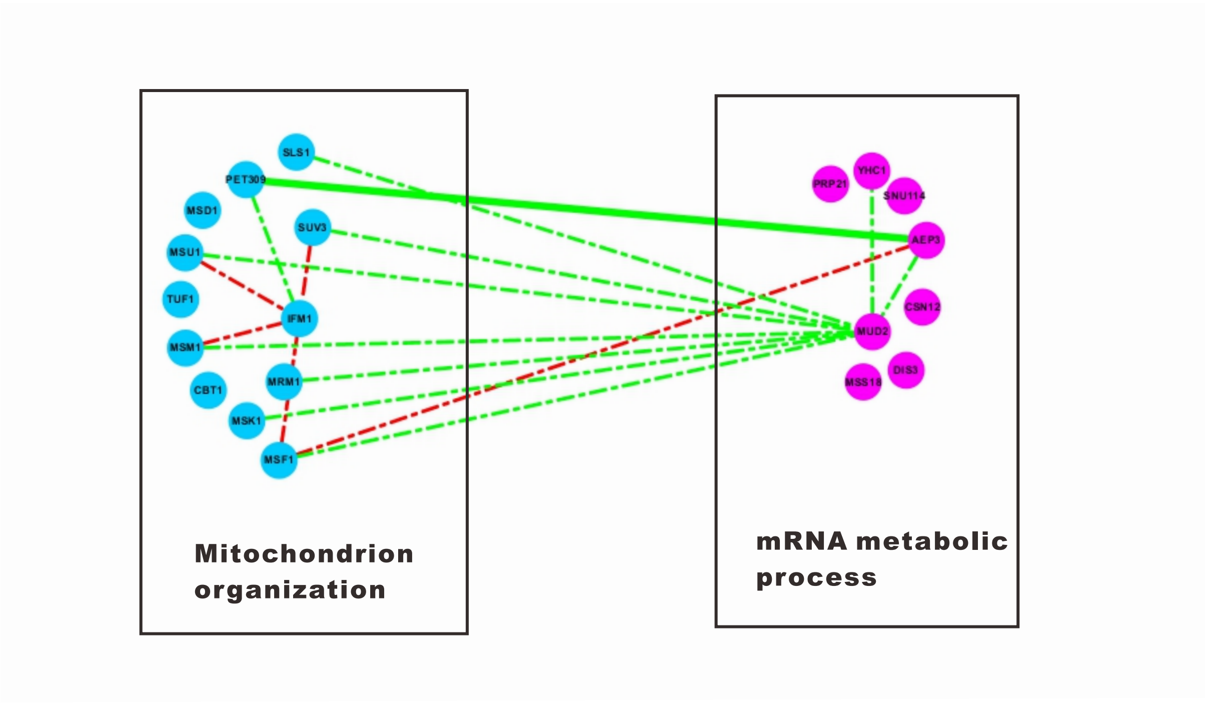

Figure 3: Global

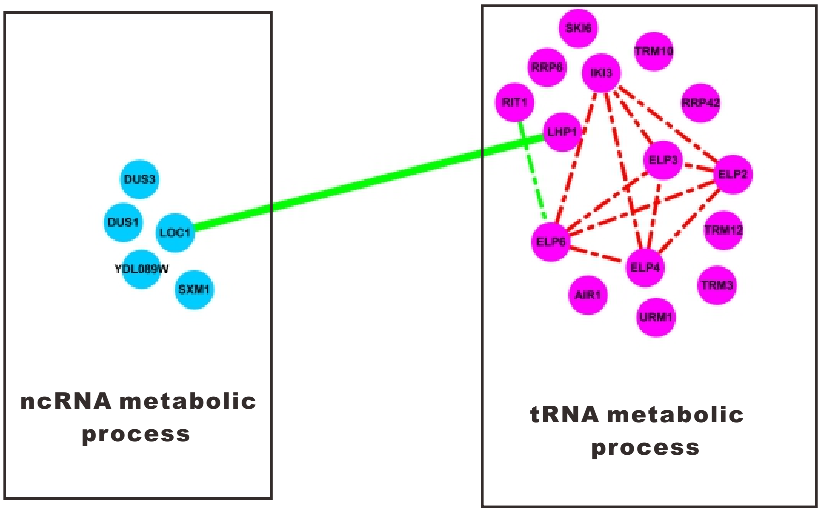

Figure 4: Global

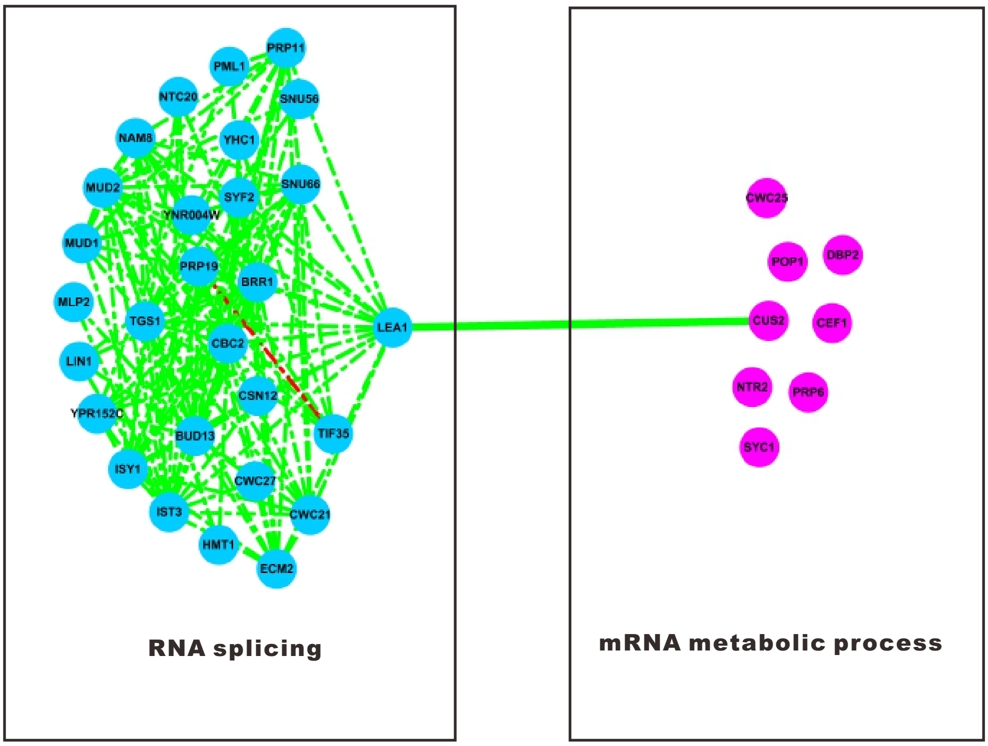

Figure 5: Global

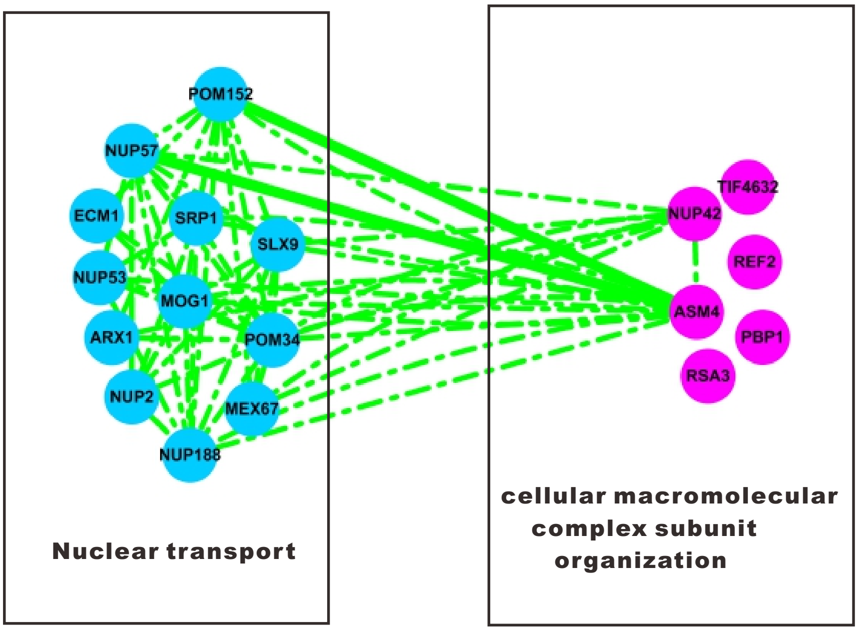

Figure 6: Global

7 Results in Synthetic data

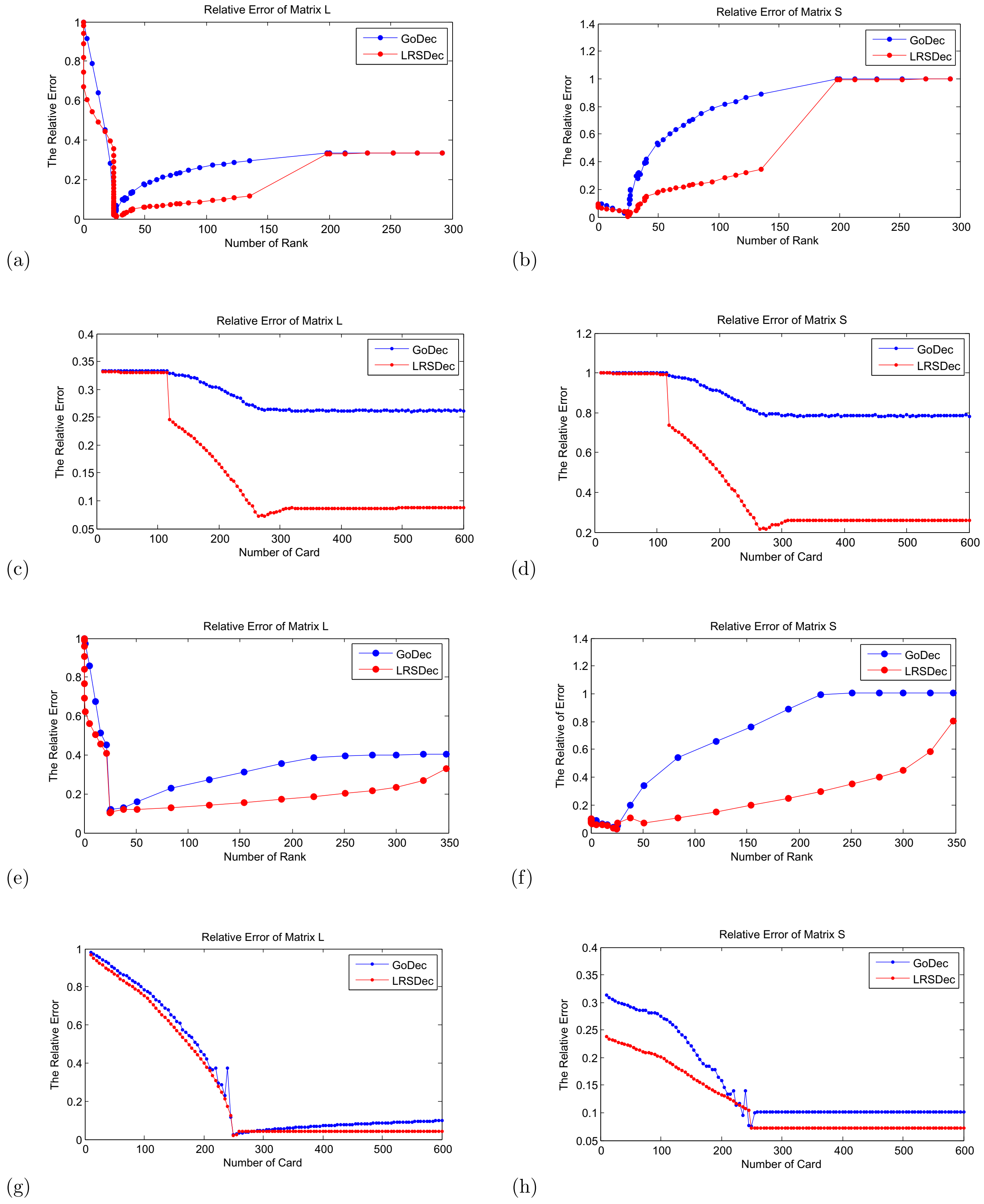

Figure 7: Performances of LRSDec and GoDec in Low-Rank and Sparse decomposition tasks on synthetic data under different paramenters. (a)-(d): nosise $\mathbf{e} = 10^{-3} * \mathbf{F}$, specially, (a)-(b): fixed parameter card, different parameter rank; (c)-(d): fixed parameter rank, different parameter card. And (e)-(h): nosise $\mathbf{e} = 10^{-1} * \mathbf{F}$, specially, (e)-(f): fixed parameter card, different parameter rank; (g)-(h): fixed parameter rank, different parameter card.

8 Application to C.elegans

Here we added the application of LRSDec algorithm to a genetic interaction dataset of Caenorhabditis elegans [1]. This dataset systematically tested genetic interactions between 11 'query' genes and 858 'target' genes. There are almost 20 % missing entries in this one. We can also get the low-rank matrix and the sparse matrix, meanwhile impute the missing entries in the dataset. We presented the results of LRSDec on this dataset in the Supplementary. After the matrix decomposition of LRSDec we could found more functional gene clusters by estimated the clustering results with GO biological process category using the hypergeometric distribution. Then if people follow our strategy 1 and 2 in the paper, they could explore the functional pathways or complexes according to the annotation dataset of *Caenorhabditis Elegans*. Furthermore, we could found more functional clusters than that in the original paper. Such as "Regulation of signal transduction", "Cell cycle switching", "Positive regulation of cellular" and so on.

Table 1: Clustering Results

♯ Clusters	low-rank matrix L		Original matrix	
	JC-Index	♯ Enriched@	JC-Index	♯ Enriched@
9	0.146	9	0.112	9
18	0.121	18	0.082	18

@: hyper-geometric test applied to test the enrichment of gene sets. Significance level :FDR<=0.05. ♯*Cluster*: the number of clusters to cut off the hierarchical clustering tree. ♯*Enriched*: the number of modules predicted by hierarchical clustering enriched in the GO iterms

References

[1] Alexandra B Byrne, Matthew T Weirauch, Victoria Wong, Martina Koeva, Scott J Dixon, Joshua M Stuart, and Peter J Roy. A global analysis of genetic interactions in caenorhabditis elegans. *J Biol*, 6(8), 2007.

[2] Adrian S Lewis and Jérôme Malick. Alternating projections on manifolds. *Mathematics of Operations Research*, 33(1):216–234, 2008.

[3] Rahul Mazumder, Trevor Hastie, and Robert Tibshirani. Spectral regularization algorithms for learning large incomplete matrices. *The Journal of Machine Learning Research*, 99:2287–2322, 2010.

[4] Jimin Song and Mona Singh. How and when should interactome-derived clusters be used to predict functional modules and protein function? *Bioinformatics*, 25(23):3143–3150, 2009.

[5] Gwendolyn M Wilmes, Megan Bergkessel, Sourav Bandyopadhyay, Michael Shales, Hannes Braberg, Gerard Cagney, Sean R Collins, Gregg B Whitworth, Tracy L Kress, Jonathan S Weissman, et al.

A genetic interaction map of rna-processing factors reveals links between sem1/dss1-containing complexes and mrna export and splicing. *Molecular cell*, 32(5):735–746, 2008.
